# A clinically authentic mouse model of enterovirus 71 (EV-A71)-induced neurogenic pulmonary oedema

**DOI:** 10.1038/srep28876

**Published:** 2016-06-30

**Authors:** Carla Bianca Luena Victorio, Yishi Xu, Qimei Ng, Beng Hooi Chua, Sylvie Alonso, Vincent T. K. Chow, Kaw Bing Chua

**Affiliations:** 1 Temasek Lifesciences Laboratory, 1 Research Link, National University of Singapore, Singapore; 2 Department of Microbiology and Immunology, Yong Loo Lin School of Medicine, National University of Singapore, Singapore; 3 Office of Research and Development, Curtin University, Perth, Western Australia, Australia

## Abstract

Enterovirus 71 (EV-A71) is a neurotropic virus that sporadically causes fatal neurologic illness among infected children. Animal models of EV-A71 infection exist, but they do not recapitulate in animals the spectrum of disease and pathology observed in fatal human cases. Specifically, neurogenic pulmonary oedema (NPE)—the main cause of EV-A71 infection-related mortality—is not observed in any of these models. This limits their utility in understanding viral pathogenesis of neurologic infections. We report the development of a mouse model of EV-A71 infection displaying NPE in severely affected animals. We inoculated one-week-old BALB/c mice with an adapted EV-A71 strain and identified clinical signs consistent with observations in human cases and other animal models. We also observed respiratory distress in some mice. At necropsy, we found their lungs to be heavier and incompletely collapsed compared to other mice. Serum levels of catecholamines and histopathology of lung and brain tissues of these mice strongly indicated onset of NPE. The localization of virally-induced brain lesions also suggested a potential pathogenic mechanism for EV-A71-induced NPE. This novel mouse model of virally-induced NPE represents a valuable resource for studying viral mechanisms of neuro-pathogenesis and pre-clinical testing of potential therapeutics and prophylactics against EV-A71-related neurologic complications.

Following the near eradication of polioviruses, EV-A71 is now considered to be the neurotrophic enterovirus that exerts the greatest impact on human health worldwide. EV-A71 is a non-enveloped, single-strand, positive-sense RNA virus member of the *Picornaviridae* family^1^. It is the major causative agent of hand, foot, and mouth disease (HFMD)—a typically mild, self-limiting childhood illness. It can escalate, however, into a severe neurological disorder presenting as either acute flaccid paralysis, aseptic meningitis, brainstem encephalitis, encephalomyelitis or neurogenic pulmonary oedema (NPE). Of these, brainstem encephalitis associated with NPE is a particular concern in affected children, since they frequently present with central nervous system (CNS) pathology and neurological sequelae that confer increased risk of mortality^2^,^3^,^4^.

Although EV-A71 had been extensively studied for the past three decades, little is known about the underlying mechanism that drives this usually benign virus to savagely attack the CNS and cause debilitating neurological illnesses. In addition, current animal models of EV-A71 infection do not recapitulate in animals the full spectrum of neurological features observed in human cases; and the underlying pathological mechanisms are of questionable clinical relevance (see Wang and Yu^5^ for a review). Consequently, the pathogenesis of fatal EV-A71-induced encephalomyelitis is still not fully understood, and efforts to identify novel therapies for severely affected patients have been extremely inadequate to date. Several research groups had attempted to reproduce the pathology of EV-A71-induced human disease in experimental animals including rhesus and cynomolgous monkeys^6^,^7^,^8^,^9^, laboratory mice^5^,^10^,^11^,^12^,^13^,^14^,^15^, and other mammals^16^,^17^,^18^,^19^,^20^. However, none of these models exhibited the key neurological features that cause the majority of EV-A71-related fatalities in humans—acute brainstem encephalitis with NPE^4^,^21^,^22^,^23^. Indeed, even those animal systems that more accurately replicated the signs of EV-A71 infection in humans were found to depend on disease mechanisms that differ substantially from those observed in human patients. For example, several post-mortem studies in the past had consistently demonstrated the restricted localization of EV-A71 antigens in CNS tissues^24^,^25^,^26^,^27^— and more recently tonsillar crypt epithelium^28^— in human cases of brainstem encephalitis and NPE. In animal models, however, the virus had also been detected in non-nervous tissues including the skeletal muscles^11^,^12^,^13^,^14^,^15^,^29^ and liver^9^.

This difficulty in reproducing the full spectrum of EV-A71-induced neurological diseases in mice springs from host restriction of the virus. EV-A71 natural infection only occurs among humans, although non-human primates can be experimentally infected. Other mammals, especially small rodents, are not susceptible to infection primarily due to the incompatibility of the virus with the receptor protein expressed on rodent cells—which had been identified recently as SCARB2 (Scavenger Receptor Class B, Member 2) protein. SCARB2 interaction with EV-A71 induces viral uncoating and subsequent cellular infection in humans^30^,^31^. Murine cells that ectopically express the human form of SCARB2 protein are completely susceptible to EV-A71 infection^32^,^33^. While there have also been efforts to create transgenic mice that express human SCARB2 protein and the other EV-A71 receptor P-selectin glycoprotein ligand-1 (PSGL-1), none of these infection models exhibits NPE^31^,^34^,^35^,^36^,^37^.

Our group generated two novel EV-A71 strains (EV71:TLLm and EV71:TLLmv; GenBank accession numbers KF514879 and KF514880, respectively) adapted through serial passage of a clinical isolate (EV71:BS) in a mouse embryonic fibroblast (NIH/3T3, ATCC CRL-1658) cell line^38^. Unlike EV-A71 clinical isolates, these adapted strains productively infected and replicated to high titres in both rodent and primate cell lines. Their genomes also accumulated multiple non-synonymous mutations, most notably in the capsid (P1) and RNA-dependent RNA polymerase (3D) genes^38^. Of these, three amino acid substitutions in VP1 protein—K98E, E145A, and L169F—that were common to both strains were demonstrated to facilitate viral infection of murine cells by enabling the virus to utilize the form of SCARB2 protein expressed on rodent cells (Victorio *et al*., 2016. *In Press*^39^). Thus, EV71:TLLm and EV71:TLLmv were the first reported EV-A71 strains that infected mice using the physiologically expressed SCARB2 protein in mouse cells.

In a bid to develop a clinically authentic mouse model of EV-A71 neurologic infection, we inoculated the adapted strains into BALB/c mice and assessed the resulting illness. We also evaluated the construct validity and face validity of the animal model by describing the underlying disease mechanisms. In addition, we used a variety of histopathological and immunohistochemical techniques to compare the virus-induced pathology in mice with observations in fatal human EV-A71 cases published in the literature.

## Results

### Infection dynamics of modified EV-A71 strains in murine hosts

In developing a clinically authentic model of neurological disease using the adapted viral strains, we first assessed the virulence of EV71:TLLm and EV71:TLLmv relative to the parental clinical isolate EV71:BS. We inoculated 10^6^ CCID_50_ (Median Cell Culture Infectious Dose) of virus either via the intraperitoneal (I.P.) or intramuscular (I.M.) route into 1-week-old mice and compared the virus-induced disease pathology.

**Figure 1:**
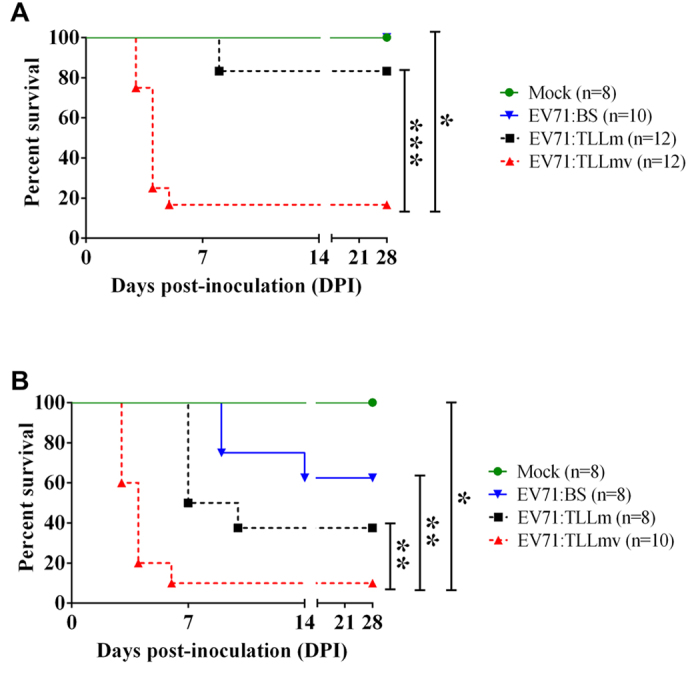
Disease severity in 1-week-old mice infected with EV71:BS, EV71:TLLm, or EV71:TLLmv. Kaplan-Meier survival curves of mice inoculated with EV71:BS, EV71:TLLm, or EV71:TLLmv via I.P. (intraperitoneal) (**A**) or I.M. (intramuscular) route (**B**). Deaths recorded were a result of disease or euthanasia at moribund stage. Mean duration of survival were compared using the Mantel-Cox log-rank test. *p < 0.05; **p < 0.005; ***p < 0.0005.

Only mice inoculated I.P. with EV71:TLLm or EV71:TLLmv progressed to lethal disease (Fig. 1A), although seroconversion was observed in all I.P.-inoculated animals (see Figure S1 in the Supporting Information). Survival rates over the 28-day observation period were 83.33% in the EV71:TLLm-infected group (10/12 mice) and 16.67% in the EV71:TLLmv-infected group (2/12 mice), whereas EV71:BS infection was uniformly non-lethal. EV71:TLLmv-inoculated mice exhibited shorter survival time compared to mice inoculated with EV71:BS (*p* = 0.0092) or EV71:TLLm (*p* = 0.0002). Similarly, we observed lower survival rates among animals inoculated I.M. with EV71:TLLm (25% survival; 2/8 mice) or EV71:TLLmv (10% survival; 1/10 mice) compared to EV71:BS (62.5% survival; 5/8 mice) (Fig. 1B). Mice inoculated I.M. with EV71:TLLmv exhibited shorter survival time relative to mice inoculated with EV71:BS (*p* = 0.0016) or EV71:TLLm (*p* = 0.0039). These findings demonstrate that of the three strains evaluated, EV71:TLLmv was associated with the greatest levels of lethality and was therefore used in all subsequent experiments.

**Figure 2:**
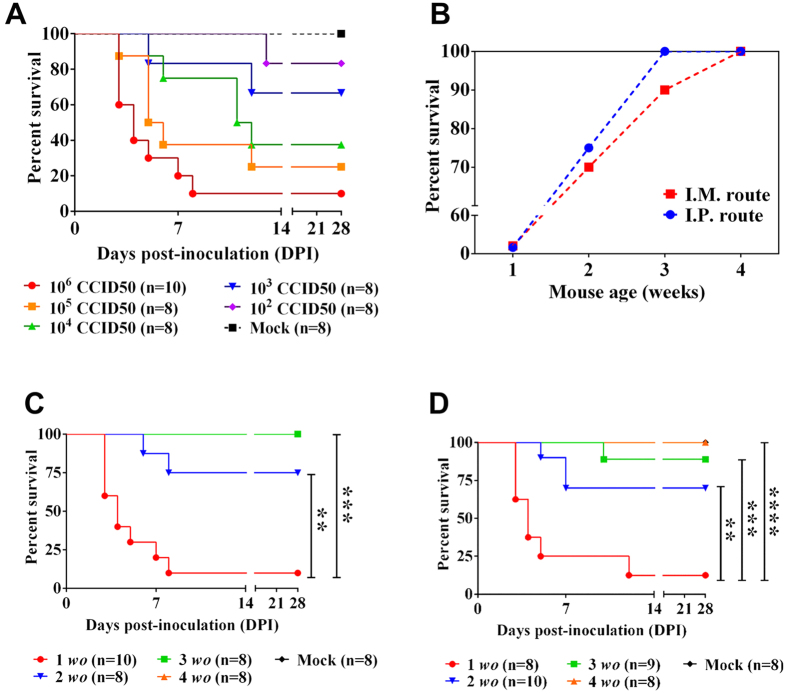
Dependence of EV71:TLLmv infection severity on the dose, age, and inoculation route. (**A**) Kaplan-Meier survival curve of 1-week-old mice inoculated I.P. with different doses of EV71:TLLmv. (**B**) Age and inoculation route dependence of survival of mice inoculated with 10^6^ CCID_50_ virus. (**C**,**D**) Kaplan-Meier survival curves of mice inoculated with 10^6^ CCID_50_ virus via I.P. (**C**) or I.M. (**D**) route. Deaths recorded were a result of disease or euthanasia at moribund stage. Mean duration of survival were compared using Mantel-Cox log-rank test. **p < 0.005; ***p < 0.0005; ****p < 0.0001. *Mock*. Sham-inoculated controls; *wo*, mouse age in weeks.

We next determined the optimal virus dose, inoculation route, and mouse age to be used for further model development. To determine the optimal dose, we inoculated 1-week-old BALB/c mice with various ten-fold serial dilutions of EV71:TLLmv via the I.P. route. Disease severity was found to vary with virus dose. The lowest survival rate (10%) was achieved in mice inoculated with 10^6^ CCID_50_ virus, and the median humane endpoint (HD_50_) was calculated to be equivalent to 3.98 × 10^3^ CCID_50_ (Fig. 2A; see Figure S2). To evaluate the effect of inoculation route and mouse age on virulence, we inoculated mice of varying ages between 1-week and 4-weeks old with 10^6^ CCID_50_ virus via either I.P. or I.M. injection. Both inoculation route and mouse age also influenced the severity of virus-induced disease (Fig. 2B). Younger animals exhibited consistently poorer prognosis than older animals—one-week-old mice inoculated I.P. exhibited lower survival rates than two-week-old mice (*p* = 0.0021) and older age groups (*p* = 0.0002) (Fig. 2C), and similar data were obtained for mice inoculated I.M. with virus (Fig. 2D). Despite the absence of clinical disease in 3-week-old mice inoculated I.P. with virus (100% survival; n = 8), some 3-week-old mice (1/9) inoculated I.M. with virus succumbed to disease. Seroconversion was also observed in all mice that survived virus inoculation, including those that did not exhibit signs of disease (see Figure S3A–C). These data reveal that EV71:TLLmv induced acute, severe infection that was lethal in mice aged 1–3 weeks old. Of the conditions tested here, disease severity was greatest in 1-week-old mice injected with 10^6^ CCID_50_ of virus either into the peritoneal cavity (I.P.) or muscle tissue (I.M.). Since I.P. inoculation more closely resembles the natural oral route of virus infection, we employed this method for all subsequent animal studies.

### Disease progression in EV71:TLLmv-infected mice

**Figure 3:**
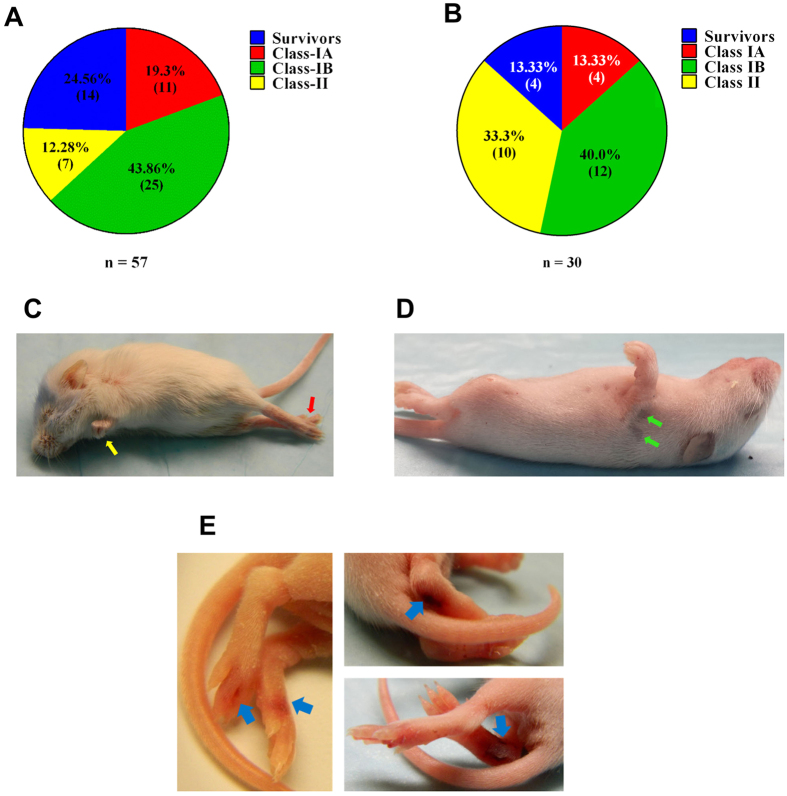
Clinical presentation of EV71:TLLmv infection in 1-week-old mice. Mice inoculated with 10^6^ CCID_50_ virus by I.P. (**A**) or I.M. (**B**) injection were grouped into four categories based on clinical signs observed throughout the observation period. Severely infected mice (both Class-I and Class-II) exhibited flaccid paralysis (**C**), either in the forelimbs (*yellow arrows*), hind limbs (*red arrows*), or both; and hairless lesions (*green arrows*) (**D**) that persisted for several days and typically disappeared within 1 week. A few Class-I mice also exhibited haemorrhagic lesions in the hind feet (**E**), which appear to be due to vasculitis (*blue arrows*).

One-week-old mice inoculated I.P. with EV71:TLLmv rapidly succumbed to disease and exhibited myriad clinical signs of neurological illnesses. Based on clinical presentation, the sick animals were categorized into four distinct groups (Table 1). ‘Survivors’ included mice that did not appear moribund at any point during the observation period (28 days). ‘Class-I’ animals presented with severe signs including an inability to self-right and altered state of consciousness—either stupor or coma—within 3–7 days post-inoculation (DPI). All mice in this group also exhibited spastic limb paresis and/or paralysis (fore-limbs, hind-limbs, or both). While some Class-I animals were devoid of respiratory signs (Class-IB), others were additionally characterized by signs of respiratory distress including tachypnea, hiccupping, gasping, and subcostal recession (Class-IA; see Movie S1, Movie S2 in the Supporting Information). Finally, ‘Class-II’ mice presented after 7 DPI with signs including persistent flaccid paralysis of the limbs (>48 h duration) and severe weight loss (>20% max. body weight). Combining the results from three independent experiments, the majority of pups inoculated I.P. with EV71:TLLmv were categorized into Class-I. Class-IA animals comprised 19.3% (11/57 mice; patent respiratory signs), while Class-IB animals comprised 43.9% (25/57 mice; no overt respiratory signs). Class-II animals represented just 12.3% (7/57) of the infected pups, and Survivors constituted 24.5% (14/57) of all infected mice (Fig. 3A). Similar distribution among the various classes of infected mice was observed when looking into the experiments individually (see Figure S4A). The combined results from two independent experiments of I.M. inoculation in mice also revealed a distribution pattern similar to the I.P-inoculated group (Fig. 3B). Majority of the inoculated mice were categorized into Class-IA (13.33%, 4/30) and Class-IB (40.0%, 12/30). However, more animals were grouped into Class-II (33.3%) and fewer animals survived (Survivors, 13.33%). This distribution was also reflected in the individual experiments (see Figure S4B). Some infected animals also presented with ataxia, tremors (localized or whole-body), unsteady gait, and limb paresis (either transient or persisting until the time of euthanasia. Of note, severely affected animals exhibited persistent limb paralysis, haemorrhagic lesions on the paws and feet due to vasculitis, and hairless lesions on the back (Fig. 3C–E). Together, these results demonstrate that mice infected with EV71:TLLmv exhibited variable incidence and severity of both neurological and respiratory symptoms that reflect the full spectrum of neurologic diseases observed in human patients, including cardio-respiratory distress.

### Neurogenic pulmonary oedema (NPE) in mice presenting Class-IA signs

**Figure 4:**
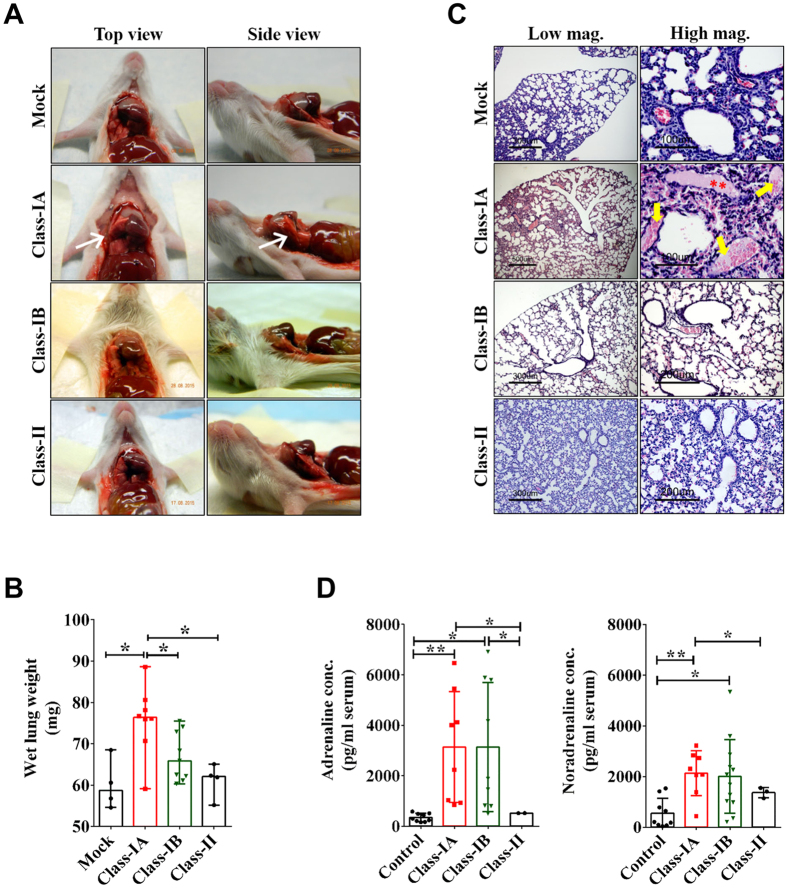
Evidence of EV71:TLLmv-induced neurogenic pulmonary oedema (NPE) in Class-IA mice. (**A**) Representative images of lungs (top- and side-views) at necropsy. Note the incomplete collapse of the lungs in Class-IA mice (*white arrows*). (**B**) Comparison of wet weights of harvested lungs. Columns represent median values (n = 4), which were compared using Mann-Whitney test. Error bars represent the range of values. *p < 0.05. (**C**) Representative tissue cross sections (5 μm) of lungs shown in low- and high-power magnifications. Note the presence of pink proteinaceous fluid in the alveolar spaces (*red asterisks*), some of which were also filled with erythrocytes (*yellow arrows*), in Class-IA lung sections. (**D**) Comparison of adrenaline (epinephrine) and noradrenaline (norepinephrine) levels in terminally collected sera. Columns represent mean values, which were compared using *t*-test with Welch’s correction for unequal variance. Error bars represent SD. *p < 0.05; **p < 0.005.

We next investigated whether the respiratory distress observed in Class-IA mice was a consequence of virus-induced pulmonary oedema (PE). Comparison of gross lung pathology between Class-IA, Class-IB, and Class-II mice revealed that Class-IA lungs were swollen, incompletely collapsed at necropsy (Fig. 4A), and displayed increased wet weight relative to lungs from either mock-infected healthy controls (*p* = 0.0162), Class-IB (*p* = 0.0206), or Class-II mice (*p* = 0.0283) (Fig. 4B). Comparison of pathology from lung tissue sections from sham-inoculated and Class-IA lungs revealed focal haemorrhage and accumulation of proteinaceous, erythrocyte-filled fluid in the alveolar spaces—features that were absent from the lungs of Class-IB and Class-II mice (Fig. 4C). Furthermore, we found no evidence of inflammatory cell infiltrate and viral antigens in the lungs and cardiac tissues collected from any group of mice (see Figure S5). These data demonstrate that the respiratory distress evident in Class-IA mice could not be attributed to either pneumonitis or congestive heart failure.

We next assessed whether the PE arising in Class-IA mice was of neurogenic origin by measuring the serum levels of catecholamines to ascertain whether neurotransmitter concentrations were modulated in EV71:TLLmv-infected mice with overt respiratory signs (Class-IA). We observed that blood concentrations of adrenaline (epinephrine) were significantly elevated in Class-IA mice compared with either Class-II (*p* = 0.0117) or mock-infected animals (*p* = 0.0086) (Fig. 4D). Similarly, serum noradrenaline (norepinephrine) levels in Class-IA mice were significantly higher than those detected in either Class-II (*p* = 0.0500) or mock-infected animals (*p* = 0.0025). These data confirm that Class-IA mice exhibited virus-induced NPE prior to death.

### Neurotropism of EV71:TLLmv in Class-IA, Class-IB, and Class-II mice

**Figure 5:**
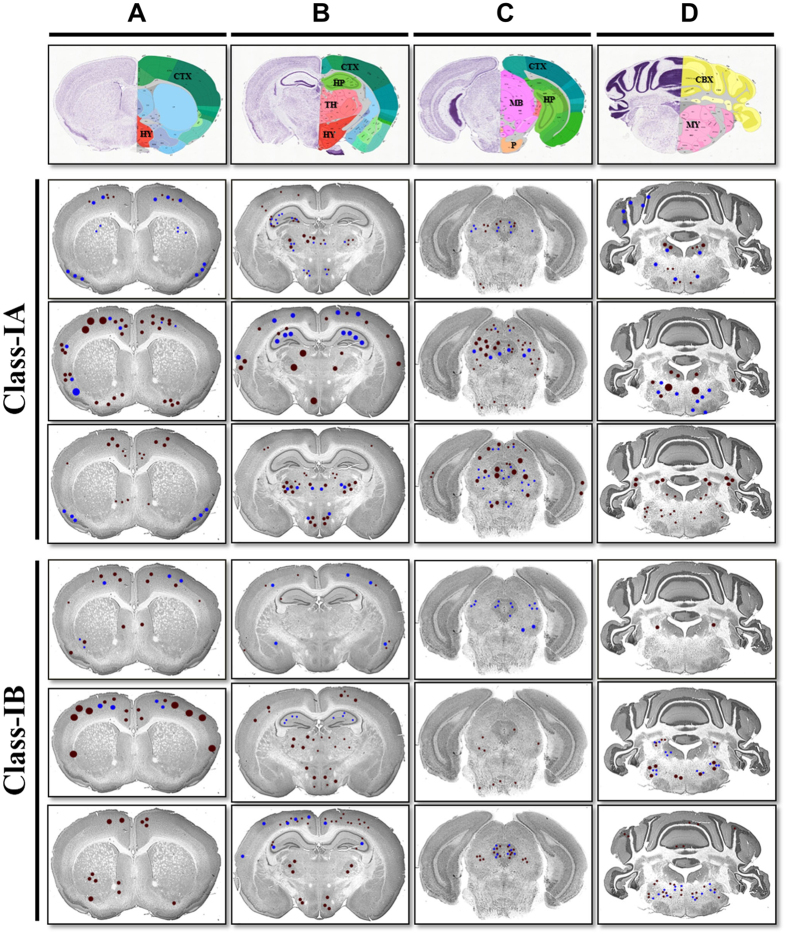
Localization and distribution of viral antigens and lesions in Class-IA and Class-IB brains. Representative images of mouse brain coronal sections marked to demonstrate the localization of viral antigens (*brown dots*) and pathological lesions (*blue dots*) observed in the brains of infected mice. Larger dots represent more intense signals or greater lesion size. Shown are the cerebellar cortex (*CTX*) (**A**–**C**); hypothalamus (*HY*) (**A**,**B**); hippocampus (*HP*) (**B**,**C**); thalamus (*TH*) (**B**); midbrain (*MB*) and pons (*P*) (**C**); and cerebellum (*CBX*) and medulla oblongata (*MY*) (**D**). Templates of coronal sections were downloaded from www.brainstars.org^58^ and http://mouse.brain-map.org/static/atlas^59^.

To determine the underlying mechanisms that might promote the development of NPE in Class-IA mice only, we assessed the distribution of virus and pathological lesions in the brains and spinal cords of animals from each disease class. The majority of animals in Class-IA and Class-IB groups exhibited ubiquitous staining of viral antigens (>10 positive neurons detected) and some pathological lesions (>5 lesions observed) in all of the CNS regions assessed (Table 2). In contrast, only 1 mouse in Class-II (n = 5) exhibited viral antigens and/or lesions in the sensory cortex, hippocampus, diencephalon, mesencephalon, medulla oblongata, or lumbar spinal cord. When we further compared CNS pathology between Class-IA and Class-IB mice, we observed a similar localization of viral antigens and tissue lesions within the hippocampus, diencephalon, mesencephalon, cerebellum, and medulla. However, the pathology was more severe in animals with Class-IA disease (Fig. 5). Indeed, when compared with Class-IB mice, animals in Class-IA group exhibited more extensive neuronal degeneration, phagocytosis, and necrosis in CA3 neurons of the hippocampus (Fig. 5B; see Figure S6A). We also observed intense viral antigen staining accompanied by marked tissue inflammation and neuronal necrosis in the Class-IA hypothalamus, whereas these pathological features were scarce in Class-IB hypothalamus (Fig. 5A,B; see Figure S6B). Compared to Class-IB brains, Class-IA brains also displayed more severe tissue pathology and viral antigen intensity in the ventro-posterior complex of the thalamus (Fig. 5B; see Figure S6C); the mesencephalon-associated tissues including the peri-aqueductal gray (PAG) matter, midbrain reticular area, and motor-related superior colliculus (Fig. 5C; see Figure S6D); as well as in the Purkinje cells and dentate nucleus of the cerebellum (Fig. 5D; see Figures S6E and S7).

In both disease groups (Class-IA and Class-IB), the most extensive distribution of viral antigens and pathological lesions involving neuronal damage and tissue inflammation were detected in the medulla oblongata (Fig. 5D; see Figure S6F), particularly in the motor-related reticular nuclei—intermediate reticular nuclei (IRN) and parvicellular reticular nuclei (PARN)—and spinal nucleus of the trigeminal nerve (sptV) at the ventrolateral regions of the caudal medulla. However, only Class-1A mice exhibited viral antigens and tissue lesions in the ventral and dorsal regions of the medullary reticular nucleus (MdRN), as well as the nucleus of the solitary tract (NTS) and area postrema (AP)—which are components of the mouse dorsal vagal complex and situated caudal to the 4^th^ ventricle (see Figure S7). For comparison, representative images of the hippocampus, hypothalamus, thalamus, midbrain, cerebellum, and medulla from mock-infected mice are also shown (see Figure S8).

**Figure 6:**
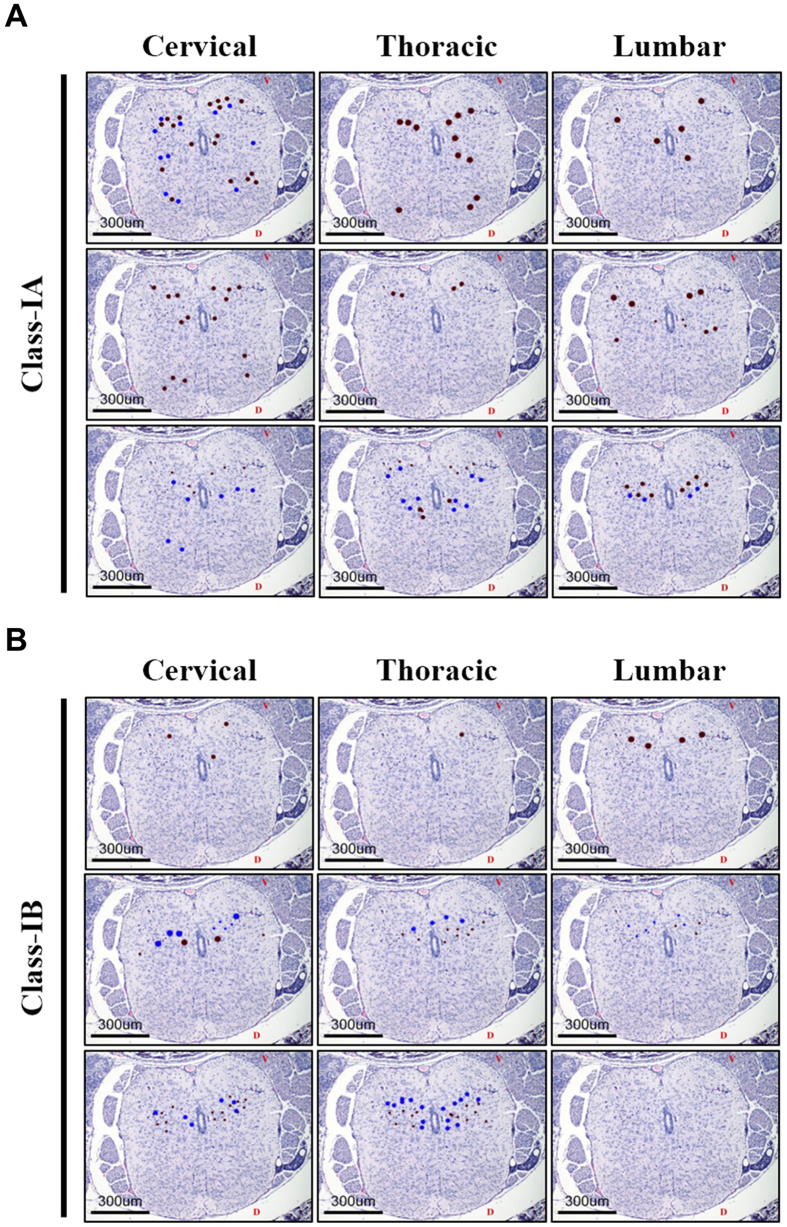
Localization and distribution of viral antigens and lesions in Class-IA and Class-IB mouse spinal cords. Representative images of spinal cord coronal sections from Class-IA (**A**) and Class-IB (**B**) mice marked to show the localization of viral antigens (*brown dots*) and pathologic lesions (*blue dots*). Larger dot sizes indicate more intense signals or lesions. *V*, ventral side; *D*, dorsal side.

In contrast, Class-IA and Class-IB mice did not differ in the distribution, localization and extent of tissue lesions and viral antigen staining within the cerebral cortex and pons (Fig. 5A,B; see Figure S9). Similarly, no difference in distribution, intensity and localization of lesions and viral antigens were observed in the spinal cord (Fig. 6); majority of which were detected in the ventral horns of the gray matter (see Figure S10). We were unable to detect viral antigens in any tissues outside of the CNS, except in the skeletal muscles of mice inoculated with EV71:TLLmv via the I.M. route (see Figure S11). These data are consistent with our hypothesis that NPE is triggered by virus-induced damage to specific brain regions rather than a uniform increase in EV-A71-related pathology across all tissues.

## Discussion

In the current report, we demonstrate that infection of 1-week-old BALB/c mice with an adapted strain of EV-A71 (EV71:TLLmv) induced a diverse repertoire of clinical signs ranging from skin lesions to severe neurological disease including paralysis, ataxia, tremors, and acute encephalomyelitis associated with NPE. Infected animals displayed varying levels of virus-induced tissue damage in different CNS regions including the pyramidal, extra-pyramidal and autonomic nervous systems, consistent with the pathology observed in fatal cases of human EV-A71 infection^24^,^25^,^26^,^27^,^40^. In mice inoculated with EV71:TLLmv via the I.P. route, we were unable to detect viral antigens or virus-induced lesions outside the CNS. In mice that received the virus by I.M. injection, however, viral antigens were present in the hind limb skeletal muscles near the site of injection only. This is consistent with previous reports that mechanical damage aids enterovirus replication in the inoculated muscle^14^,^41^,^42^.

Based on disease presentation at the time of euthanasia, infected mice could be readily classified into four distinct groups: Class-IA, Class-IB, Class-II and Survivors. While the Survivors did not present any signs of disease, Class-II mice exhibited persistent flaccid paralysis and severe weight loss requiring euthanasia, and Class-IA and Class-IB mice additionally suffered from acute neurological disease that was universally lethal within 3–7 DPI. Class-IA mice also exhibited acute severe respiratory distress and gross pathological observations revealed incomplete lung collapse at necropsy and significantly increased lung wet weight relative to other groups. In addition, histopathological analyses revealed focal haemorrhage and accumulation of proteinaceous, erythrocyte-laden transudate in the alveolar spaces. These features closely resembled those observed in non-virally-induced NPE in experimental animal models and human fatal cases^23^,^29^,^43^,^44^,^45^,^46^.

Pulmonary oedema (PE) could be defined as an extravascular increase in the water content of the lungs and subcategorized on the basis of cardiogenic or neurogenic origin. Class-IA heart tissues exhibited normal histology and lacked overt signs of disease, so we were able to exclude cardiogenic PE. Class-IA lungs also lacked evidence of viral replication and inflammation in the lung parenchyma, so direct virus-induced tissue injury could be excluded. Instead, Class-IA mice exhibited elevated serum levels of catecholamines, strongly suggesting that the respiratory distress observed in these mice were signs of NPE—which had been previously identified as a consequence of catecholamine storm induced by severe sympathetic discharge^43^,^47^. In this scenario, excessive release of catecholamines promotes systemic and pulmonary vasoconstriction, leading to a shift in blood volume from the systemic to the pulmonary circulation, and culminating in plasma leakage and haemorrhage into the alveolar spaces due to hydrostatic disruption or direct injury to the pulmonary endothelium^47^,^48^,^49^,^50^. Although PE could also be induced by host cytokine storm in response to EV-A71 infection^51^,^52^, our data strongly suggest that a major mechanism of EV-A71-induced NPE did not involve a massive systemic inflammatory response but rather associated with tissue damage in specific regions of the brain. This concept was also supported by findings in previous experimental animal models of non-virally induced NPE^43^,^53^,^54^.

NPE induction had also been previously associated with certain brain regions such as the hypothalamic paraventricular and dorsomedial nuclei^47^,^49^; and the ventrolateral and dorsal medulla—which include the nucleus of the solitary tract (NTS) and area postrema (AP)^43^,^50^,^53^,^54^,^55^. The NTS, AP, and dorsal motor nucleus of the vagus nerve (DMX) form interconnected structures in the dorsal vagal complex. The NTS integrates sensory information received from visceral afferent fibers and passes information to the dorsal motor nucleus of the vagus nerve (DMX), which subsequently sends parasympathetic signals to visceral organs, particularly to the cardiovascular and respiratory systems. Post-mortem findings on human fatal cases of EV-A71-induced NPE also suggested the involvement of these areas in triggering NPE^23^,^24^,^26^,^56^. In our mouse model, abundant viral antigens and extensive tissue damage were widely distributed throughout the brainstem and spinal cord tissues of Class-IA and Class-IB mice. Moreover, serum catecholamine levels were significantly elevated in both Class-IA and Class-IB compared with sham-inoculated controls. In Class-IA mice, severe destruction of the NTS, seemed to be the main contributing factor to the development of NPE. Our results suggest that the absence of NPE in Class-IB mice may be explained by lesser extent of damage and tissue destruction in known NPE trigger zones—NTS, medullary reticular nuclei (MdRN), and AP regions of the medullary tissues; cerebellar dentate nucleus; and dorsomedial nuclei of the anterior hypothalamic tissues. We therefore propose that acute, severe destruction of brainstem tissue leads to a catecholamine storm, which progresses to NPE if known trigger zones in the brainstem become sufficiently damaged by the virus.

This proposed potential pathogenic mechanism for EV71-induced NPE leads to further questions about the neuro-pathogenesis of EV-A71. Firstly, is the consistent observation of the virus in specific regions of the brainstem, rather than uniformly throughout the tissue, due to either virus tropism for certain cell types or non-uniform susceptibility of the available cells? Secondly, what is the role of the virus receptors SCARB2 and PSGL-1, if any, in the neuro-pathogenesis of EV-A71? Third, what are the molecular determinants that enable EV71:TLLmv to induce NPE in mice?

In summary, we have developed a clinically authentic mouse model of EV-A71 neuro-infection that exhibits genuine face validity^5^. Mice infected with EV71:TLLmv display the full spectrum of clinical signs and neurological features that are induced by EV-A71 infection in human patients—most importantly NPE. Our model also displays construct validity^5^ with respect to both gross and histopathological features, which closely resemble those reported in fatal human disease. Although several experimental animal models of non-virally-induced NPE have been developed in recent years (see Sedy^49^ and Davison^50^ for review), our model is the first to successfully induce the classical signs of NPE using EV-A71 infection alone. This new model represents a powerful tool for identifying key events in EV-A71 neurologic pathogenesis, dissecting mechanisms of EV-A71-induced NPE, and developing novel treatment modalities for severely affected patients. Future studies will also be able to exploit this model to conduct pre-clinical evaluation of novel therapeutic agents and methods of prophylactic protection against EV-A71.

## Materials and Methods

### Ethics statement

The number of animals used and procedures employed were reviewed and approved by the Institutional Animal Care and Use Committee (IACUC) of Temasek Lifesciences Laboratory (TLL-IACUC approval no. TLL-14-023), which follows the guidelines setup by the National Advisory Committee on Laboratory Animal Research (NACLAR) of Singapore. Images and video recordings were also obtained with approval from TLL-IACUC. Neurological examination of inoculated animals was performed following the guidelines and standard procedures set by The Institutional Care and Use of Animals Committee (IACUC) of University of California, San Francisco^57^.

### Mouse and virus strains

Adult specific pathogen-free (SPF) BALB/c mice were purchased from InVivos, Inc. (Singapore), and mated to obtain pups. All mice were maintained within the Animal Facility of Temasek Lifesciences Laboratory and housed in Individually-Ventilated Cages (IVC) lined with clean wood shavings and supplemented with cotton nesting material. Mice were also provided with food pellets and distilled water *ad libitum*, and were allowed to mate to obtain newborn mice. EV-A71 strains used for inoculation (EV71:BS, EV71:TLLm, and EV71:TLLmv) had been described and characterized previously^38^. Virus stocks of known titer and matching lot were stored at −80 °C and thawed at 4 °C before use. Stocks were pre-warmed to room temperature immediately prior to animal inoculation.

### Study design

An *a priori* statistical power test was conducted via a Mann-Whitney comparison of two groups (two-tailed α = 0.05) performed in G*Power3 statistical software^58^ to determine the appropriate number of animals to allocate to each treatment group. Each cage litter containing a dam and 8–10 pups was randomly assigned to the various treatment groups. In cages where litter size was <8 pups, the animals were transplanted into age-matched litters at 2–3 days post-partum. Inoculated mice were followed for 28 days thereafter; body weight and general health status were assessed twice daily during the first week post-inoculation and then once daily during subsequent weeks. Animals that become moribund were euthanized immediately by intraperitoneal (I.P.) injection of pentobarbitone (90 mg/kg). Criteria for euthanasia of moribund animals complied with previously set guidelines^13^: (1) loss of >20% maximum recorded body weight, (2) paralysis lasting >48 h, (3) absence of feeding or inability to feed, (4) inability to self-right, and (5) altered state of consciousness presenting as either stupor or coma. Blood and tissue samples were collected at necropsy (except for animals that died during the night or were cannibalized, and therefore excluded from the study). Animals that survived the 28-day observation period were sacrificed by pentobarbitone injection as described above, and terminal blood collection was performed by cardiac puncture with a 26G needle to facilitate analysis of neutralizing antibody titers in serum.

In order to develop a clinically authentic model, we first compared the virulence of the adapted strains EV71:TLLm and EV71:TLLmv with the parental clinical isolate EV71:BS.

We inoculated groups of 6-day-old mice (n = 8–10 per group) with the maximum 10^6^ cell culture infective dose (CCID_50_) by I.P. injection and then observed for disease presentation and animal survival. We then determined the optimum mouse age, virus dose, and inoculation route to be used in the study. Groups of 8–10 mice of various ages (1, 2, 3, or 4 weeks old) were inoculated I.P. with the maximum dose of EV71:TLLmv (10^6^ CCID_50_), while groups of 6-day-old BALB/c mice (n = 8–10 per group) were inoculated I.P. with various doses of virus (10^6^, 10^5^, 10^4^, 10^3^, 10^2^, 10^1^, 0 CCID_50_). To compare the effects of inoculation route, we infected groups of 6-day-old BALB/c mice (n = 8–10 per group) with 10^6^ CCID_50_ by either I.P. or I.M injection.

### Animal handling and infection

Virus inoculations were performed inside a biosafety cabinet (Class II) using a 31G ultra-fine, hub-less insulin syringe (Beckton, Dickinson and Company, Franklin Lakes, New Jersey, USA) to reduce animal distress. Mice were acclimatized for 5 min prior to handling, inoculation, or observation.

### Necropsy, gross pathological observations, and tissue collection

Euthanized animals were necropsied inside a Class II biosafety cabinet using standard protocols to harvest organs. Gross pathologic examination was also performed and photographs were taken for record. Lungs were superficially flushed twice with sterile PBS and then blotted dry on filter paper prior to measuring wet weight. Organs harvested for histological studies were stored in 10% neutral buffered formalin (NBF) for 1 week at 4 °C.

### Tissue processing for histological analyses

Fixed tissues were dehydrated in a series of increasing concentrations of ethanol (70%, 95% and 100%). Tissues were incubated in two changes of alcohol and three changes of Histoclear II (Electron Microscopy Sciences, Hatfield, Philadelphia, USA), then finally infiltrated with four changes of melted paraffin wax. All incubations were performed for 1 h at room temperature with gentle rocking at 100 rpm. Paraffin infiltrations were performed in an oven set at 65 °C. Paraffin-embedded tissue blocks were sectioned (5 μm) using a microtome, loaded onto poly-lysine-coated glass slides, dried overnight at 42 °C, then stored at room temperature until further use.

### Staining of tissue sections

Tissue sections were de-waxed by incubation in two changes of Histoclear II and then slowly rehydrated in decreasing alcohol concentrations (100%, 95%, 70%, and 50%). Slides were incubated in PBS for 10 min prior to staining. Hematoxylin and Eosin (H&E) staining was performed by first flooding the slides with Harris’ Hematoxylin (Sigma Aldrich, St. Louis Missouri, USA) and incubating at room temperature (RT) for 15 min. The slides were then rinsed in water, de-stained in 1% acid alcohol (95% ethanol, 1% HCl), dipped in 0.2% NH_4_OH, and rinsed in water for 10 min prior to counterstaining in eosin solution. The slides were next de-stained in 95% ethanol, dehydrated by three changes of absolute alcohol and two changes of Histoclear II. Tissues were finally set in DPX mounting fluid (Sigma Aldrich, St. Louis Missouri, USA).

### Immunohistochemistry

Following de-waxing and rehydration, slides were subjected to heat-induced antigen retrieval by incubation in a histology-grade BP-125 microwave oven (Microwave Research and Applications, Inc., Carol Stream, Illinois, USA) and citrate buffer (pH 6.0) for 30 min at 96 °C. Slides were allowed to cool to RT over 3 h and were subsequently blocked with 5% normal pig serum (Sigma Aldrich, St. Louis Missouri, USA) for 1 h at RT. Without further washing, the slides were then incubated at 4 °C overnight in rabbit serum containing polyclonal antibodies against EV-A71 (generous gift from Dr. Hiroyuki Shimizu of the National Institutes of Infectious Diseases in Tokyo, Japan). The slides were then washed 5× in Tris-buffered saline (pH 7.4), 0.05% Tween-20 (TBS-T), and rinsed 2× in TBS prior to quenching endogenous peroxidases by addition of 3% H_2_O_2_ for 1 h at RT. Slides were subsequently washed 2× in TBS prior to incubation with swine anti-rabbit Ig-HRP (Dako Cytomation, Glostrup, Denmark) for 1 h at RT. After washing, slides were incubated in diaminobenzidine (DAB) substrate, and counterstained with Hematoxylin.

### Mapping viral antigens and virus-induced lesions in tissue sections

Template images of representative coronal sections of the mouse brain were downloaded from www.brainstars.org^59^. These images are free to be used and modified under license from the Creative Commons of Japan. The appearance and location of lesions and viral antigens were observed under the microscope and marked onto corresponding areas of the template images. Affected brain regions were identified using the mouse brain atlas of coronal sections (www.mouse.brain-map.org/static/atlas)^60^. Similarly, a representative coronal section of the thoracic spinal cord was used as a template onto which the location of lesions and viral antigens was mapped.

### Measurement of serum catecholamine levels

Blood samples were collected by cardiac puncture of moribund animals during necropsy. Serum was obtained by centrifuging coagulated blood samples at 3000 × *g*, 4 °C, for 30 min, then stored at −20 °C until determination of catecholamine levels using the 2-CAT (A-N) ELISA kit (Labor Diagnostika Nord, Nordhorn, Germany) according to the manufacturer’s protocol.

### Measurement of neutralizing antibody levels in sera from infected mice

Blood samples were collected by cardiac puncture at necropsy. The blood was clotted at room temperature for 30 min and then centrifuged for 20 min at 3000× g, 4 °C to obtain sera. Samples were stored at −20 °C until further analysis. Randomly selected samples of frozen sera were assayed in neutralization tests. Dulbecco’s Modified Eagle’s Medium (MEM) supplemented with (1% ^v^/_v_) Fetal Bovine Serum (FBS) was used to perform 2-fold serial dilutions of the neat sera (1:20 to 1:1280) into 96-well plates prior to mixing with 100 CCID50 virus. The mixture was then incubated for 1 h at 37° C before addition of murine embryonic fibroblast NIH/3T3 (ATCC CRL-1658) cells (6,000 cells/well). Plates were incubated at 37 °C and observed for signs of CPE between days 4–10. Neutralizing antibody titers were determined using the Reed and Muench method (reported as units per ml sera).

### Statistical analyses

All graphs were created and statistical analyses performed using GraphPad Prism version 6.01 for Windows (GraphPad Software, La Jolla, California, USA; www.graphpad.com). Normal distribution of data was assessed using Q-Q plots and Kolmogorov-Smirnov tests. The mean values of normally distributed data were compared by *t*-test (with Welch’s correction for datasets displaying unequal variance). The median values of non-normally distributed data were compared using Mann-Whitney tests. Comparison of Kaplan-Meier survival curves was performed using Mantel-Cox log-rank tests.

## Additional Information

**How to cite this article**: Victorio, C. B. L. *et al*. A clinically authentic mouse model of enterovirus 71 (EV-A71)-induced neurogenic pulmonary oedema. *Sci. Rep*. **6**, 28876; doi: 10.1038/srep28876 (2016).

## Supplementary Material

Supplementary Information

Supplementary Movie S1

Supplementary Movie S2

## Figures and Tables

**Table 1:** Clinical features of EV71:TLLmv-infected BALB/c mice at the time of euthanasia.

	Class-IA	Class-IB	Class-II	Survivors^a^
Time to onset of signs [days post-infection; DPI]	Within 3–5 DPI	Within 3–7 DPI	>7 DPI	N/A^b^
Level of consciousness	Stupor^c^/Coma^d^	Stupor^c^	Active	Active
Weight loss	<20% body weight	<20% body weight	>20% body weight	None or <20% body weight
Limb function (>48 h)	Spastic limb paresis/paralysis	Spastic limb paresis/paralysis	Flaccid limb paralysis	Normal
Breathing function	Tachypnea /gasping/hiccupping	Regular	Regular	Regular
Cardiac function	Tachycardia	Tachycardia^e^/Regular	Regular	Regular
Limb tremors	May be present	May be present	Absent	Absent

^a^Animals were assessed at the end of the observation period (28 DPI).

^b^Not Applicable.

^c^Animal was unable to self-right, but responded to physical stimulation of the toes.

^d^Animal was unable to self-right and did not respond to physical stimulation of the toes.

^e^Tachycardia was observed in 35% of Class IB mice.

**Table 2:** CNS distribution of viral antigens and virus-induced lesions in terminally-ill mice.

		Class-IA				Class-IB				Class-II			
		EV-A71 antigens		Pathology		EV-A71 antigens		Pathology		EV-A71 antigens		Pathology	
Cerebral cortex	Motor cortex	+++^a^	100%^b^ (5/5)	+++^c^	80%^d^ (4/5)	+++	80% (4/5)	+++	80% (4/5)		0% (0/5)		0% (0/5)
	Sensory cortex	++	60% (3/5)	++	80% (4/5)	++	80% (4/5)	++	80% (4/5)	+	20% (1/5)	+	20% (1/5)
Hippocampus		++	60% (3/5)	+	60% (3/5)	+	40% (2/5)	+	20% (1/5)	+	20% (1/5)	+	20% (1/5)
Diencephalon	Thalamus	+++	100% (5/5)	+++	60% (3/5)	++	60% (3/5)	++	20% (1/5)	+	20% (1/5)	+	20% (1/5)
	Hypothalamus	+++	80% (4/5)	+++	80% (4/5)	++	60% (3/5)	++	60% (3/5)	+	20% (1/5)	+	20% (1/5)
Mesencephalon		+++	100% (5/5)	+++	100% (5/5)	+	60% (3/5)	++	60% (3/5)	+	20% (1/5)	+	20% (1/5)
Metencephalon	Cerebellum	++	80% (4/5)	++	80% (4/5)	+	40% (2/5)	+	60% (3/5)		0% (0/5)		0% (0/5)
	Pons	++	80% (4/5)	++	80% (4/5)	++	20% (1/5)	++	60% (3/5)		0% (0/5)		0% (0/5)
Myelencephalon	Medulla	+++	100% (5/5)	+++	100% (5/5)	++	40% (2/5)	++	60% (3/5)	+	20% (1/5)	+	20% (1/5)
Spinal cord	Cervical	++	100% (5/5)	++	100% (5/5)	++	80% (4/5)	++	60% (3/5)		0% (0/5)		0% (0/5)
	Thoracic	++	100% (5/5)	++	100% (5/5)	++	80% (4/5)	++	80% (4/5)		0% (0/5)		0% (0/5)
	Lumbar	++	100% (5/5)	++	100% (5/5)	++	80% (4/5)	++	60% (3/5)	+	20% (1/5)	+	20% (1/5)

^a^Density of viral antigens detected per slide: +, <10 positive neurons; ++, 10–20 positive neurons; +++, >20 positive neurons.

^b^Percentage of animals exhibiting viral antigens in the specified brain region (n = 5).

^c^Distribution of pathologic lesions in nervous tissues: +, <5 lesions; ++, 5–10 lesions; +++, >10 lesions.

^d^Percentage of animals exhibiting lesions in the specified brain region (n = 5).
