# Supplementary material for: A clinically authentic mouse model of enterovirus 71 (EV-A71)-induced neurogenic pulmonary oedema
Source: Sci Rep. 2016 Jun 30;6:28876. doi: 10.1038/srep28876 (PMC4928123; doi:10.1038/srep28876)
Supplement: Supplementary Information [file srep28876-s1.pdf]

Supporting Information for

A clinically authentic mouse model of enterovirus 71 (EV-A71)-induced neurogenic pulmonary oedema

Authors: Carla Bianca Luena Victorio, Yishi Xu, Qimei Ng, Beng Hooi Chua, Sylvie Alonso, Vincent T.K. Chow*, Kaw Bing Chua*

*To whom correspondence should be addressed. E-mail: VTC (vincent_chow@nuhs.edu.sg); KBC (chuakb@tll.org.sg)

This file includes:

Movie S1. Hallmark observations in EV71:TLLmv-infected mice presenting Class-IA signs of disease

Movie S2. Hallmark observations in EV71:TLLmv-infected mice presenting Class-IB signs of disease

Figure S1. Seroconversion in mice inoculated (I.P.) with various EV-A71 strains

Figure S2. Median humane endpoint (HD_{50}) of EV71:TLLmv in one-week-old mice

Figure S3. Seroconversion observed in animals inoculated with EV71:TLLmv

Figure S4. Reproducible distribution of EV71:TLLmv-infected mice into different classes

Figure S5. Absence of viral replication and inflammation in the lung and heart tissues of Class-IA mice

Figure S6. Lesions and viral antigens observed in brain tissue sections of EV71:TLLmv-infected mice

Figure S7. Viral antigen and lesion localization in NPE trigger zones in the caudal brainstem

Figure S8. Representative images of brain coronal sections from mock-inoculated healthy mice

Figure S9. Comparable histopathology and viral antigen distribution between Class-IA and Class-IB mice brains

Figure S10. Lesions and viral antigens observed in spinal cord sections of EV71:TLLmv-infected mice

Figure S11. Viral replication in the limb muscles evident in I.M.-inoculated mice

SUPPLEMENTARY VIDEOS

Movie S1

Hallmark observations in EV71:TLLmv-infected mice presenting Class-IA signs of disease

This video is comprised of two video clips from two different Class-IA mice. Both animals were unable to self-right and were in a state of coma. Severe respiratory distress presenting as tachypnea with subcostal recession is evident in the first mouse. Gasping, subcostal recession and a frothy fluid emanating from the nostrils can be seen in the second mouse.

Movie S2

Hallmark observations in EV71:TLLmv-infected mice presenting Class-IB signs of disease

The animal shown in the video prior to necropsy was unable to self-right and was in a state of stupor. Ipsilateral paralysis of the right limbs and persistent tremor of the left hind-limb were also observed.

SUPPLEMENTARY FIGURES

Figure S1

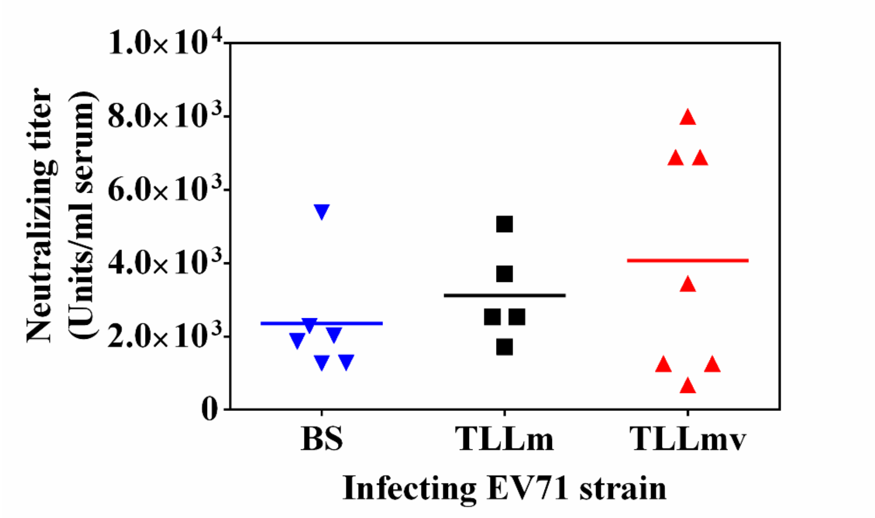

Seroconversion in mice inoculated (I.P.) with various EV-A71 strains

Neutralizing antibody titers in terminally collected sera from 1-week-old mice inoculated with different virus strains. Virus neutralization tests were performed using Vero cells for assessment of neutralization titers against EV71:BS; and NIH/3T3 cells for assessment of neutralization titers against EV71:TLLm and EV71:TLLmv. Points represent individual titre values, while lines indicate mean titre values.

Figure S2

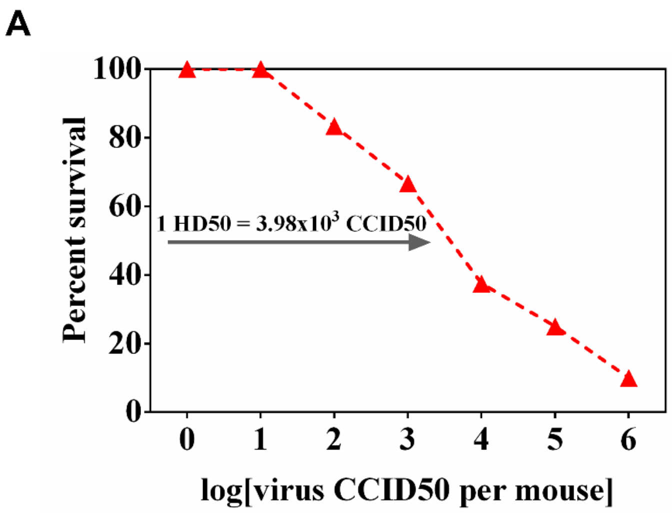

Median humane endpoint (HD_{50}) of EV71:TLLmv in one-week-old mice

The HD50 value, which refers to the virus dose that induces signs requiring euthanasia in half of the inoculated animals, was determined by inoculating (I.P.) mice with varying doses of EV71:TLLmv. The calculated HD_{50} value was equivalent to a virus dose of 3.98×10^3 $CCID_{50}$

Figure S3

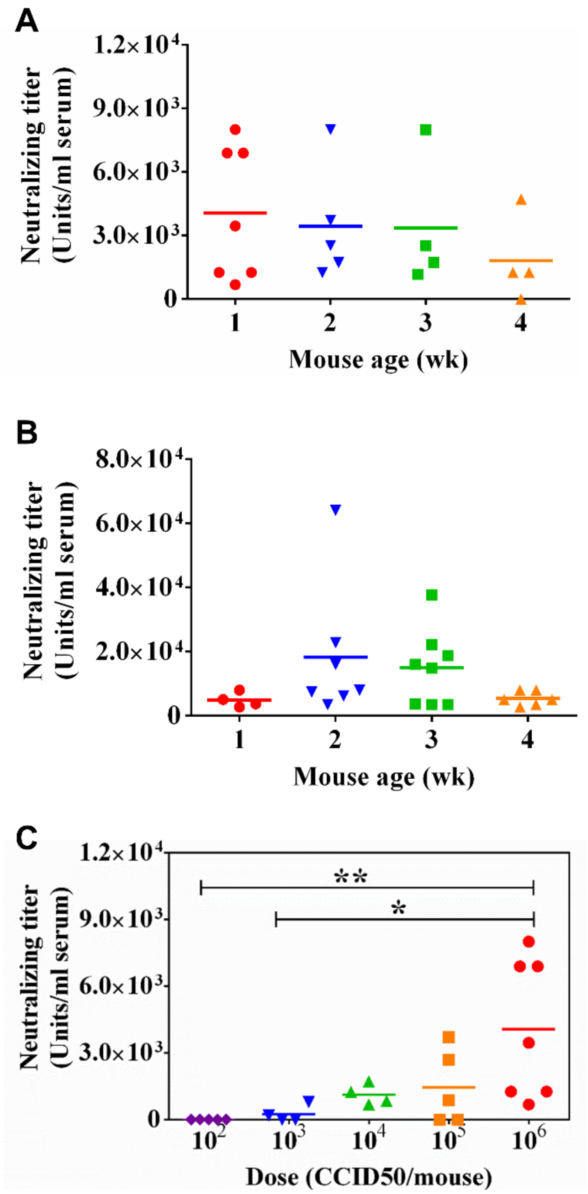

Seroconversion observed in animals inoculated with EV71:TLLmv

(**A-B**) Neutralizing antibody titres in terminally collected sera from mice of variouse ages (1-4 weeks) inoculated with 10^6 $CCID_{50}$ virus either via I.P (**A**) or I.M. (**B**) injection. (**C**) Neutralizing antibody titres in terminally collected sera from 1 week-old-mice inoculated (I.P.) with various doses of virus. Mean titre values were compared using *t*-test. * p<0.05; ** p<0.005.

Figure S4

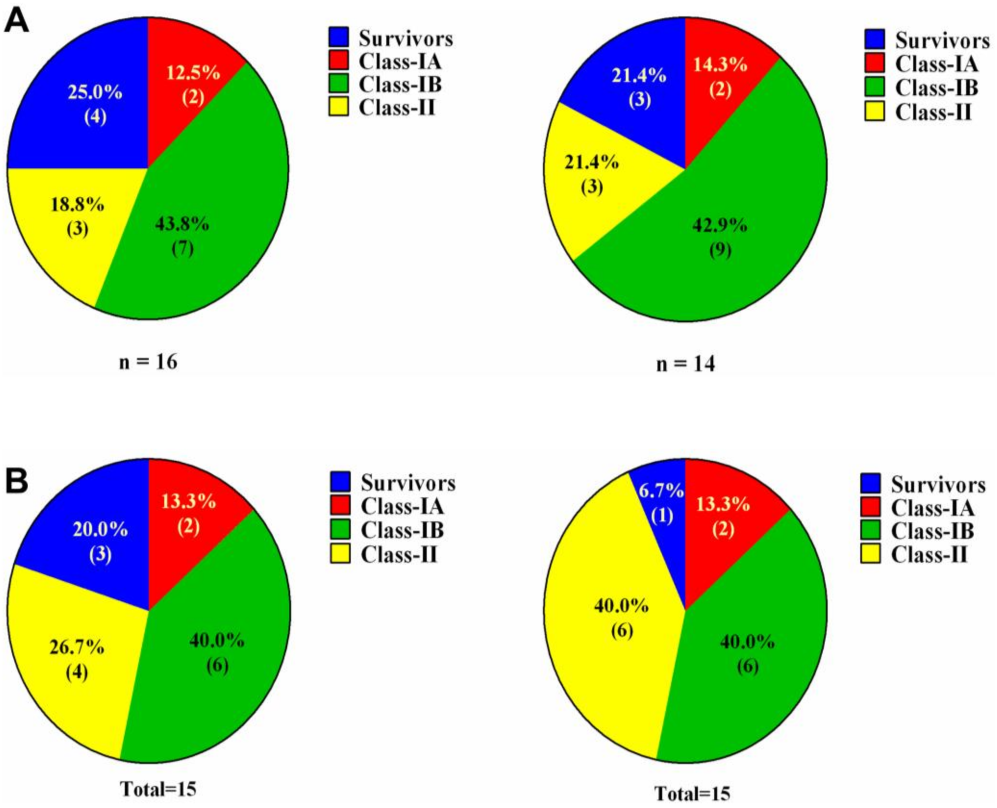

Reproducible distribution of EV71:TLLmv-infected mice into different classes

Distribution of mice inoculated either (**A**) I.P. or (**B**) I.M. with EV71:TLLmv into various classes of clinical presentation derived from two independent experiments. Each experiment was comprised of mice collected from two separate litters.

Figure S5

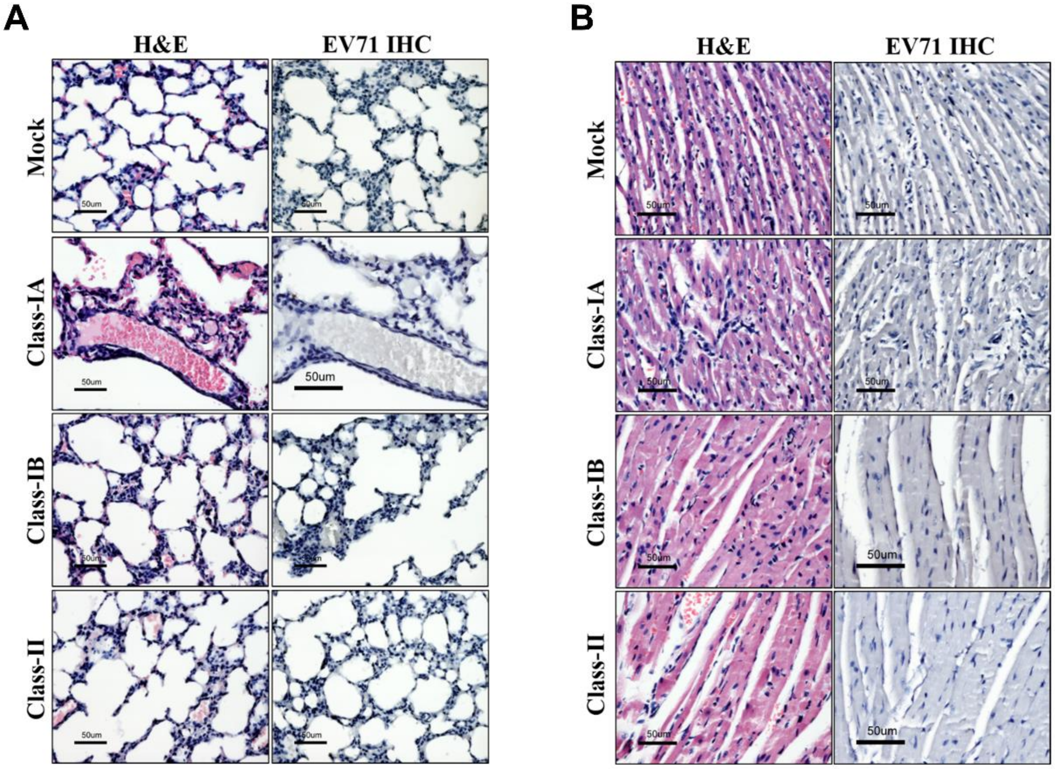

Absence of viral replication and inflammation in the lung and heart tissues of Class-IA mice

Representative images of lung tissue cross sections (5µm) (**A**) and heart tissue longitudinal sections (5µm) (**B**) stained with Hematoxylin & Eosin (H&E) for histopathological examination; or labelled with anti-EV-A71 serum (EV71 IHC) for virus antigen localization. *Mock,* control tissues from sham-inoculated mice.

Figure S6

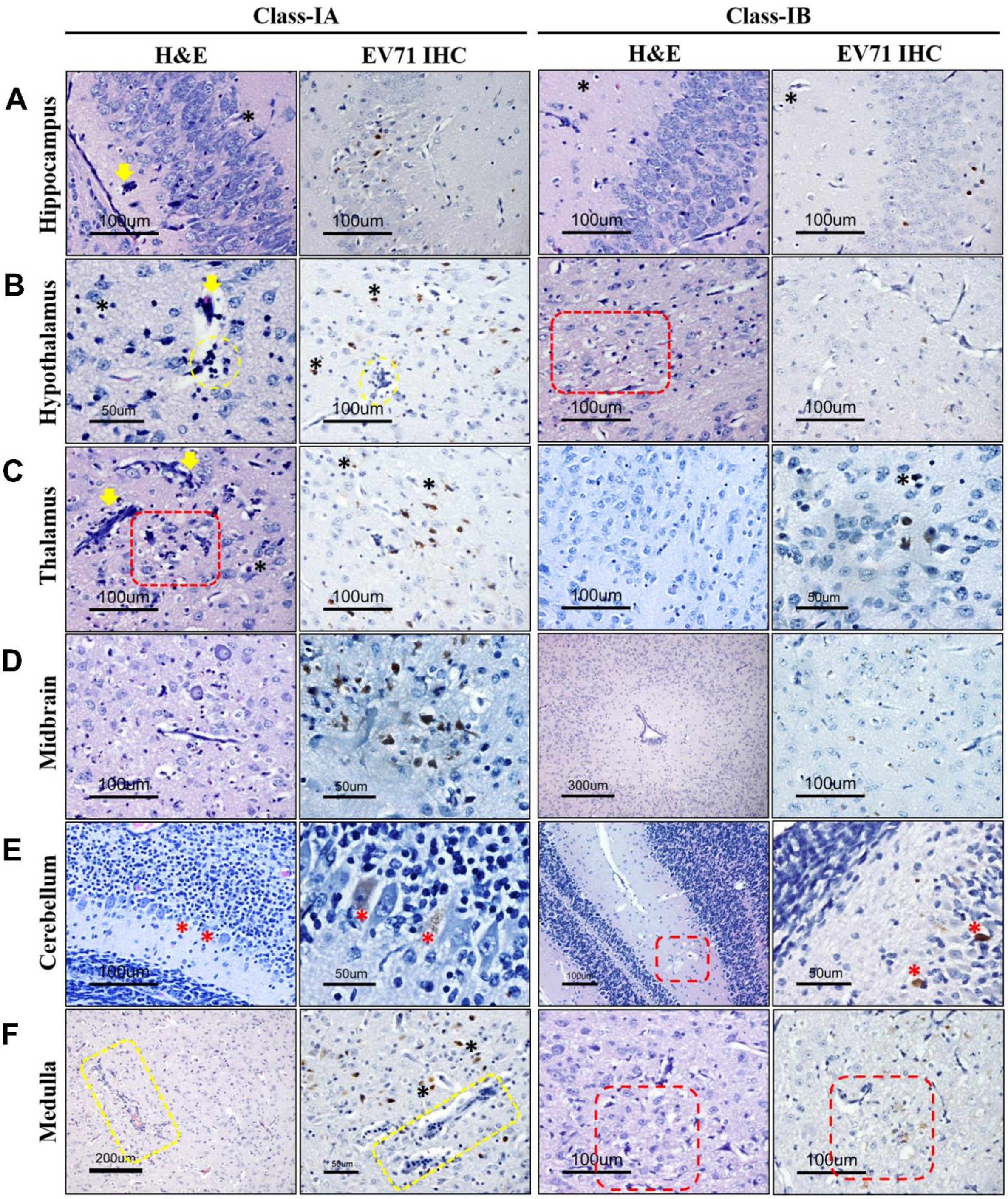
Class-IA
Class-IB
H&E
EV71 IHC
H&E
EV71 IHC
A
Hippocampus
B
Hypothalamus
C
Thalamus
D
Midbrain
E
Cerebellum
F
Medulla
100um
50um
300um
200um

Lesions and viral antigens observed in brain tissue sections of EV71:TLLmv-infected mice

Representative images of brain coronal sections (5µm) of hippocampus (**A**), hypothalamus (**B**), thalamus (**C**), midbrain (**D**), cerebellum (**E**), and medulla (**F**) derived from Class-IA (left panels) and Class-IB (right panels) mice. Sections were stained with Hematoxylin & Eosin (H&E) for histopathological examination; or labelled with anti-EV-A71 sera (EV71 IHC) for virus antigen localization. Pathological lesions observed include edema (*red box*), infiltrating immune cells (*yellow box*), neuronophagia (*yellow arrows*), neurodegeneration (*black asterisks*), and degeneration of Purkinje cells (*red asterisks*).

Figure S7

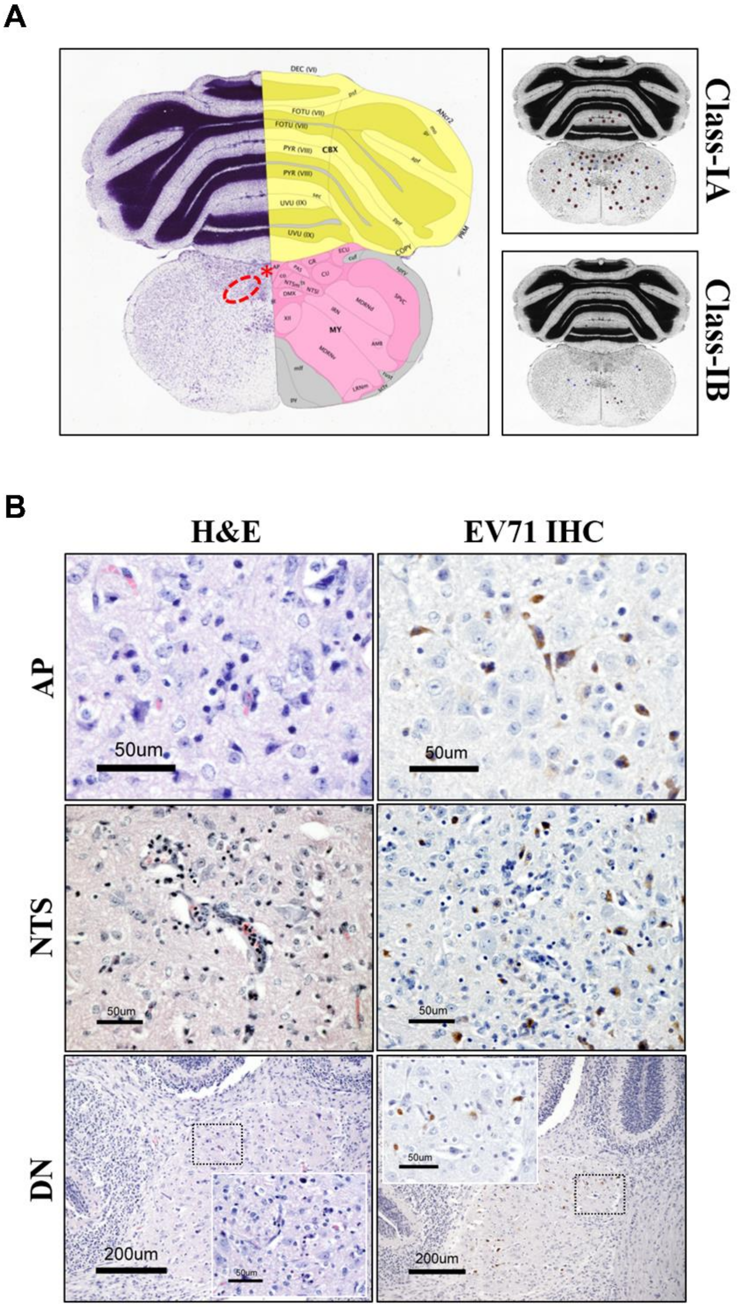

Viral antigen and lesion localization in NPE trigger zones in the caudal brainstem

(**A**) Schematic diagram of a coronal section through the caudal brainstem (*left panel*) marked to exhibit the localization of viral antigens (*brown dots*) and lesions (*blue dots*) in Class-IA and Class-IB brains (*right panels*). Template images were downloaded from The Mouse Brain Atlas and depict the location of the AP (*red asterisk*) and NTS (*red circle*). (**B**) Representative images of coronal sections through the caudal brainstem of Class-IA mice depicting the AP (area postrema), NTS (nucleus of the solitary tract), and DN (dentate nucleus). Tissue sections (5 μm) were stained with Hematoxylin & Eosin (H&E) for histopathological examination, or labelled with anti-EV-A71 sera (EV71 IHC) for virus antigen localization. Boxed areas are shown magnified in the inset. *CBX*, cerebellar cortex; *MY*, medulla oblongata.

Figure S8

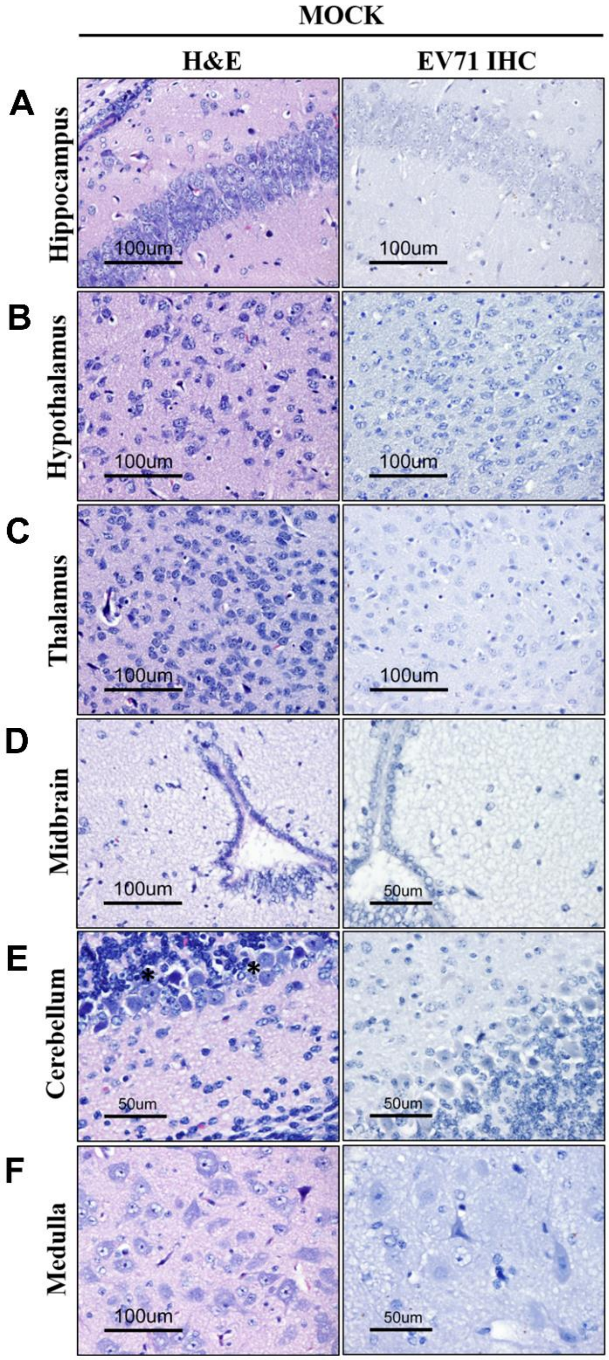
MOCK
H&E
EV71 IHC
A
Hippocampus
B
Hypothalamus
C
Thalamus
D
Midbrain
E
Cerebellum
F
Medulla
100um
50um

Representative images of brain coronal sections from mock-inoculated healthy mice

Coronal sections (5µm) of hippocampus (**A**), hypothalamus (**B**), thalamus (**C**), midbrain (**D**), cerebellum (**E**), and medulla (**F**) derived from healthy control mice sacrificed at the same day as Class-IA and Class-IB mice. Tissues were stained with Hematoxylin & Eosin (H&E) for histopathological examination; or labelled with anti-EV-A71 sera (EV71 IHC) for virus antigen localization. Note the regular morphology of Purkinje cells (*black asterisks*).

Figure S9

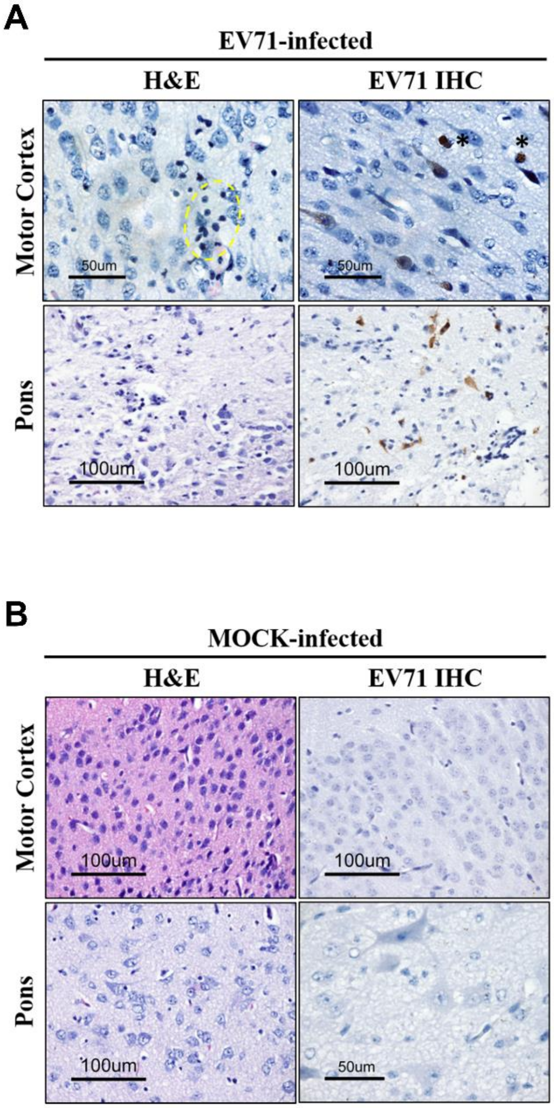

Comparable histopathology and viral antigen distribution between Class-IA and Class-IB mice brains

Coronal sections (5µm) of motor cortex and pons derived from either Class-IA or Class-IB (**A**) or mock-infected control mice (**B**). Sections were stained with Hematoxylin & Eosin (H&E) for histopathological examination; or labelled with anti-EV-A71 sera (EV71 IHC) for virus antigen localization. Note the presence of cellular infiltrate (*yellow circle*) and neuronal necrosis (*black asterisks*).

Figure S10

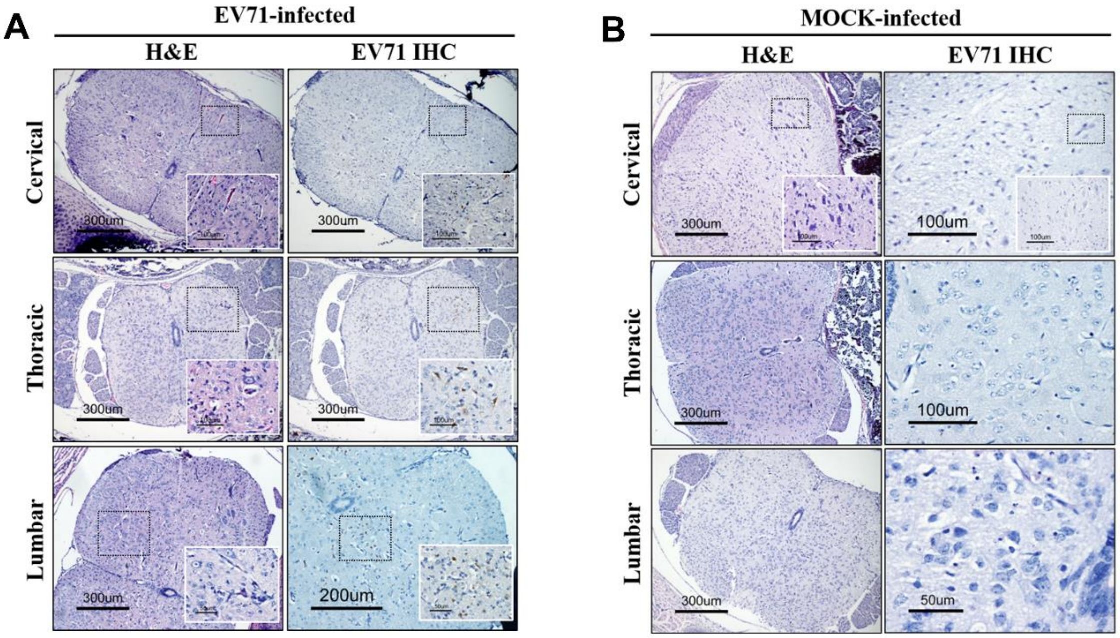

Lesions and viral antigens observed in spinal cord sections of EV71:TLLmv-infected mice

Representative images of coronal sections (5µm) of various spinal cord regions collected from either Class-IA or Class-IB mice (**A**) or mock-infected animals (**B**). Sections were stained with Hematoxylin & Eosin (H&E) for histopathological examination; or labelled with anti-EV-A71 sera (EV71 IHC) for virus antigen localization. Boxed areas are shown magnified in the respective insets.

Figure S11

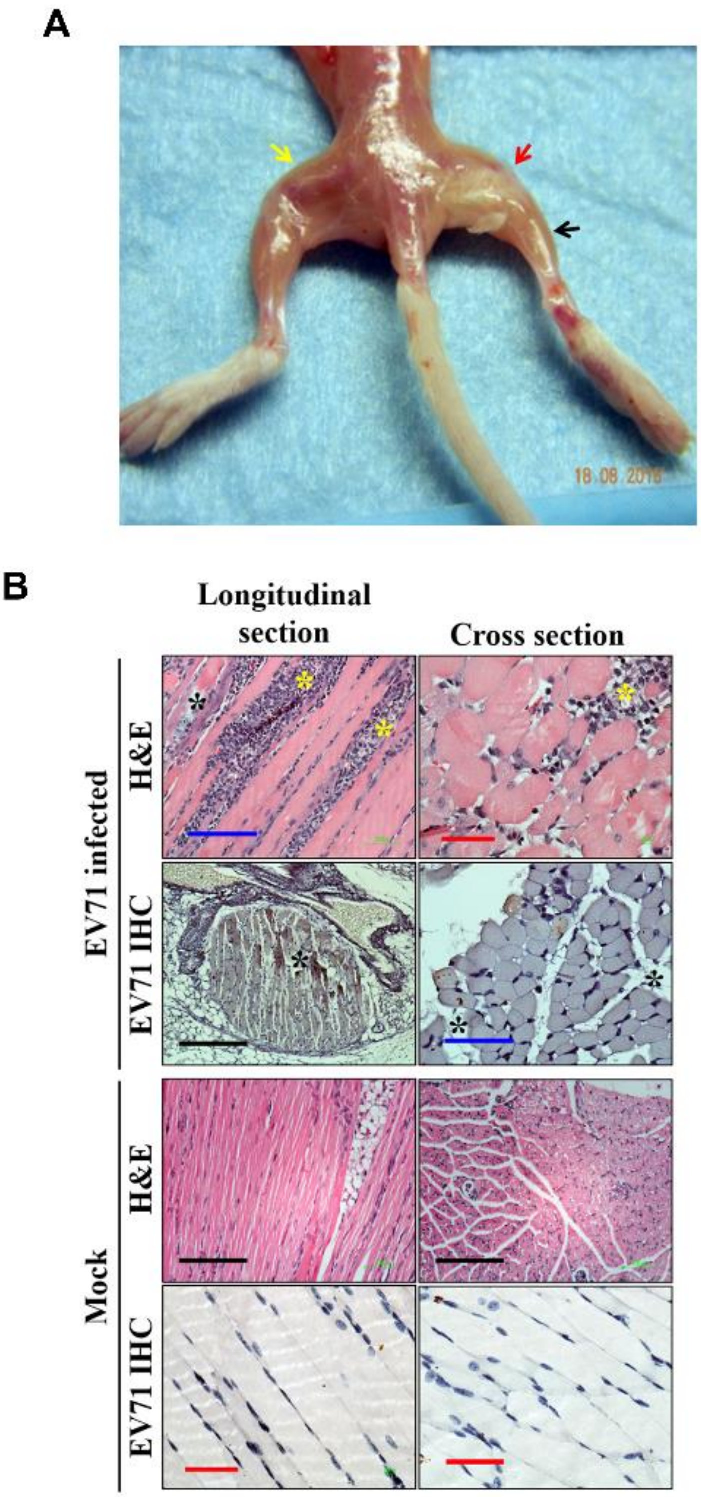
A
B
Longitudinal section
Cross section
EV71 infected
H&E
EV71 IHC
Mock
H&E
EV71 IHC

Viral replication in the limb muscles evident in I.M.-inoculated mice

(**A**) Representative image of hind limb (HL) gross pathology at necropsy revealing muscle necrosis (*red arrow*) in the right thigh above the inoculation site (*black arrow*) in comparison to normal muscle tissue (*yellow arrow*). (**B**) Representative images of transverse and longitudinal tissue sections (5µm) of the right HL of infected and sham-infected (Mock) control mice. Sections were stained with Hematoxylin & Eosin (H&E) for histopathological examination, or labelled with anti-EV-A71 sera (EV71 IHC) for virus antigen localization. Note evidence of severe inflammation with massive leukocyte infiltration (*yellow asterisks*) and muscle necrosis (*black asterisks*). Images are shown at different magnifications; Black bar=200µm; Blue bar=100µm; Red bar=50µm.
